# Fabrication of a drug delivery system that enhances antifungal drug corneal penetration

**DOI:** 10.1080/10717544.2018.1461278

**Published:** 2018-04-16

**Authors:** Jingguo Li, Zhanrong Li, Zhen Liang, Lei Han, Huayang Feng, Siyu He, Junjie Zhang

**Affiliations:** Henan Key Laboratory of Ophthalmology and Visual Science, Henan Eye Hospital, People’s Hospital of Zhengzhou University, Zhengzhou, P. R. China

**Keywords:** Fungal keratitis, corneal penetration, polymeric vector, pharmacokinetics, dual fluorescent label

## Abstract

Fungal keratitis (FK) remains a severe eye disease, and effective therapies are limited by drug shortages and critical ocular barriers. Despite the high antifungal potency and broad spectrum of econazole, its strong irritant and insolubility in water hinder its ocular application. We designed and fabricated a new drug delivery system based on a polymeric vector for the ocular antifungal application of econazole. This novel system integrates the advantages of its constituent units and exhibits superior comprehensive performance. Using the new system, drug content was significantly increased more than 600 folds. The results of *in vivo* and *in vitro* experiments demonstrated that the econazole-loaded formulation exhibited significantly enhanced corneal penetration after a single topical ocular administration, excellent antifungal activity, and good tolerance in rabbits. Drug concentrations and ocular relative bioavailability in the cornea were 59- and 29-time greater than those in the control group, respectively. Following the topical administration of one eye drop (50 μL of 0.3% w/v econazole) in fungus-infected rabbits, a high concentration of antimycotic drugs in the cornea and aqueous humor was sustained and effective for 4 h. The mechanism of corneal penetration was also explored using dual fluorescent labeling. This novel drug delivery system is a promising therapeutic approach for oculomycosis and could serve as a candidate strategy for use with various hydrophobic drugs to overcome barriers in the treatment of many other ocular diseases.

## Introduction

Fungal keratitis (FK) is a devastating ocular disease that often causes ocular morbidity and blindness, particularly in developing countries (Wu et al., 2015). Because fungi can neither penetrate the intact corneal epithelium (ICE) nor enter the cornea via vessels, the major pathogenic factors of FK include post-traumatic infection, extended wear of contact lenses, corneal surgery, chronic keratitis, and immunosuppressive diseases (Xie et al., 2006; Kaur & Kakkar, 2010). The most common fungi that cause eye infections are filamentous fungi (such as *Aspergillus fumigatus*) and yeast-like fungi (such as *Candida albicans*) (Ahn et al., 2011). FK morbidity has exhibited a clearly increasing trend in recent years, particularly in developing countries. Due to the possibility of serious infection diffusion and blindness, an effective treatment must be administered immediately upon confirmation of the diagnosis. However, current therapies against FK are often inefficient due to drug insensitivity and resistance, lack of drug availability, and lack of an ideal ocular drug delivery system (Liu et al., 2013). Natamycin suspension is the only available antifungal drug approved by the U.S. Food and Drug Administration to treat FK (Bhatta et al., 2012). Current clinical drugs also include azole compounds (such as fluconazole) and polyenes (such as amphotericin B); however, the poor penetration, stability, and solubility of these drugs have limited their application in FK (Ganegoda & Rao, 2004). Drug treatment for FK thus remains challenging and risky, and the development of broad-spectrum, efficient antifungal drugs, and novel drug delivery systems is critically needed.

Econazole (ECZ), an imidazole antifungal agent, is a potent broad-spectrum antifungal agent that is effective against many mycotic fungal infections (Mura et al., 2001). ECZ has been widely used to treat serious, invasive fungal infections of the skin, hair, and mucous membranes in clinical settings. ECZ is administered by oral or intravenous infusion to treat systemic fungal infections or as a topical cream to treat fungal skin infections (Faucci et al., 2000). Unfortunately, ECZ has very low water solubility (approximately 5 µg/mL at 25 °C), which affects the preparation of pharmaceutical formulations and limits its therapeutic applications and bioavailability (Mura et al., 1999). The solubility and preparation technology of the base compound can be improved via the formation of a salt with an acid, such as a nitrate salt, in a variety of pharmaceutical formulations. However, ECZ nitrate causes severe irritation, which drastically limits its topical application, particularly in ophthalmic cases (Hermawan et al., 2010). Topical particulate carriers for ECZ have been investigated to address this issue and promote penetration across the biological barrier to infection sites. These carriers include cyclodextrin (CD), liposome solid lipid nanoparticles, and nanostructured lipid carriers (Sanna et al., 2007). ECZ is a powerful therapeutic agent against sensitive fungal pathogens; however, there is no appropriate pharmaceutical formulation for ocular delivery due to its irritating properties and unique ocular barriers (Oji & Clayton, 1982).

**Figure 1.**
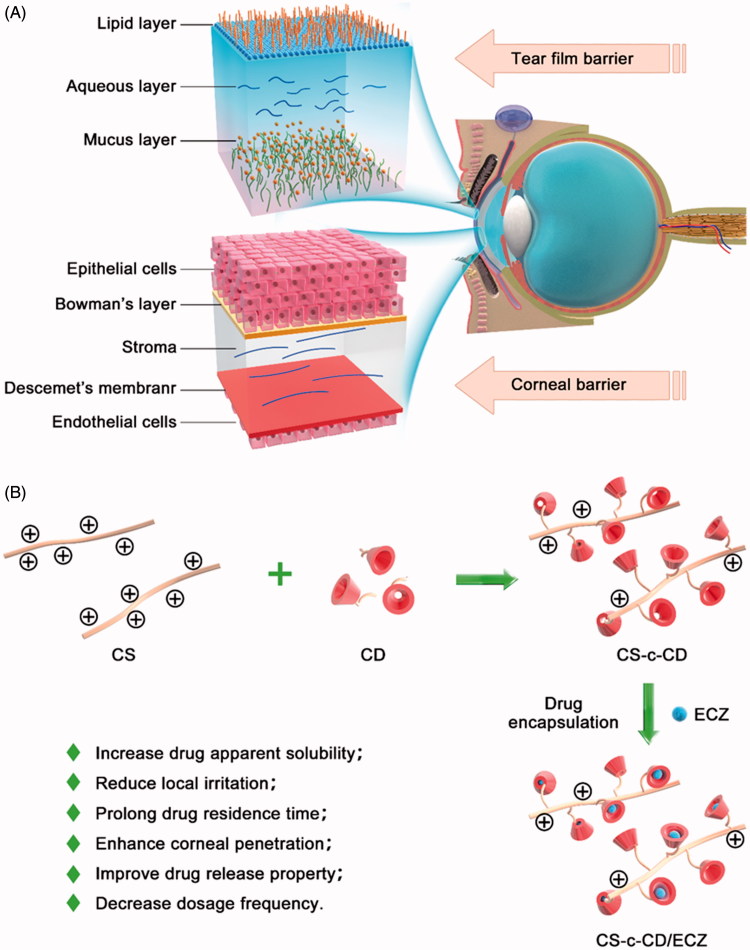
(A) Two major barriers to topical ocular drug delivery. Tear film barrier: a high tear turnover rate and gel-like mucus layer. Corneal barrier: tight junctions in the corneal epithelial cells and alternating polarity five-layer structure. (B) Schematic illustration of vector synthesis, drug loading, and the virtues of NDDS for ocular topical administration.

Due to the complex physiology and structure of the eye, three critical ocular barriers restrict the entry of drug molecules into the target site, including the tear film barrier, corneal barrier, and blood-retinal barrier (Kambhampati & Kannan, 2013). Consequently, ocular drug delivery is one of the most interesting and challenging endeavors faced by pharmaceutical scientists (Alvarado et al., 2015). For anterior segment diseases, such as infections, inflammation, and glaucoma, transcorneal penetration is the major delivery route used for optimal drug absorption (Diebold & Calonge, 2010). A noninvasive delivery system for topical ocular drugs, eye drops have always been considered the preferred route for treating anterior segment-related diseases because of their simplicity, safety, and level of patient acceptance. However, this method suffers from low bioavailability of the administered drug, as <5% reaches the aqueous humor (Urtti, 2006). To maintain and deliver a therapeutic concentration to the target site, eye drops must be administered frequently. Unfortunately, frequent topical application can cause irritation, side effects, and poor patient compliance (Jain et al., 2010). The low bioavailability of ophthalmic drugs used in eye drop formulations is attributable to two major intrinsic barriers to topical ocular drug delivery: the tear film barrier and the corneal barrier (Gan et al., 2013) (Figure 1(A)). The tear film barrier has a high tear turnover rate and is made up of a gel-like mucus layer, whereas the corneal barrier contains tight intercellular junctions among corneal epithelial cells as well as characteristics opposite of those of the cornea, including a lipophilic epithelium and a hydrophilic stroma (Baba et al., 2011). To overcome barriers and enhance ocular drug bioavailability, the main prerequisite is prolonged preocular residence of the drug delivery system and enhanced corneal permeability (Li et al., 2015). Therefore, the development of novel topical administration formulations that enhances the ocular penetration of eye drops remain one of the most challenging tasks in ophthalmic drug delivery (Mannermaa et al., 2006).

To acquire satisfactory antifungal efficacy, an ideal drug delivery system following a topical administration formulation should possess a number of attributes: (1) broad-spectrum antifungal activity; (2) superior corneal penetration; (3) a high drug concentration; (4) prolonged retention time in the precorneal area; (5) improved patient compliance with easy application, minimal side effects, and decreased frequency of administration; and (6) cost effectiveness (economical). A formulation capable of satisfying all these criteria has not yet been developed.

Here, we designed and fabricated a new drug delivery system (NDDS) to enhance the ocular penetration of eye drops and improve the antifungal efficacy of ECZ. This NDDS improves antifungal pharmacokinetics via the sustained release and prolonged retention of a high ECZ concentration localized at the precorneal sites, thus enhancing bioavailability and therapeutic efficacy. The platform presented in this study exhibits excellent corneal penetration and antifungal efficiency and might be a promising candidate for use in therapeutic eye drops, particularly those for the treatment of FK.

## Materials and methods

### Materials

Chitosan (CS, viscosity average molecular weight 30 kDa, degree of N-deacetylation 75–85%) was purchased from Jinan Haidebei Marine Bioengineering Co., Ltd. (China) and was used as received. Sodium chloroacetate, α-cyclodextrin (α-CD), N-hydroxysuccinimide (NHS), 1-ethyl-3-(3-dimethylaminopropyl) carbodiimide (EDC), and malic acid were purchased from Sigma-Aldrich (St Louis, MO, USA) and used without further purification. Cyanine 5 (Cy5) NHS ester was purchased from Lumiprobe Corporation (Hallandale Beach, FL, USA). Coumarin 6 (C6) was purchased from J&K Scientific Ltd. (Beijing, China). Dialysis bags (MW cutoff: 3.5 and 14 kDa) were purchased from Shanghai Green Bird Technology Development Co., Ltd. (Shanghai, China), and stored in 1 mM ethylenediaminetetraacetic acid aqueous solution prior to use. Econazole nitrate (ECZ·HNO_3_) was purchased from Hefei Bomei Biotechnology Co., Ltd. (China), and ECZ base was prepared from ECZ nitrate as previously described (Pedersen et al., 1998). Methanol [high-performance liquid chromatography (HPLC) grade] was purchased from Tedia Company (Fairfield, CT, USA). Injection water was used to prepare the eye drop solution. All other reagents were of analytical grade and were used as received.

### Synthesis and characterization of conjugating polymer

Carboxymethyl-α-cyclodextrin (CD-COOH) was synthesized as previously reported with slight modifications (Prabaharan & Mano, 2005). Briefly, a mixture of α-CD (9.73 g, 10 mmol) and sodium hydroxide (7.2 g, 180 mmol) in 30 mL of distilled water was treated with sodium chloroacetate (1.165 g, 10 mmol) at 50 °C for 5 h. The reaction mixture was then adjusted to pH 6.6 by adding HCl. After neutralization, the obtained product was precipitated with an excess amount of mixed solvent of methanol and acetone, filtered, and dried under a vacuum for 24 h until a constant weight was attained. The substitution degree of carboxymethyl groups on the primary hydroxyl sites of the α-CD was estimated at 14% using ^1^H NMR.

The coupling reaction of α-CD onto chitosan was performed in the presence of EDC and NHS in water at pH 7.0. Approximately 0.587 g of chitosan was dissolved in 30 mL of distilled water at room temperature for 30 min. Next, 3.093 g of α-CD-COOH (3 mmol) and 0.379 g of NHS (3.3 mmol) were dissolved in 20 mL of distilled water, and then EDC (0.63 g, 3.3 mmol) was added. The reaction mixture was stirred for 2 h and then slowly added to the chitosan solution. The reaction mixture was stirred at room temperature for an additional 24 h. The system was centrifuged and the supernatant solution was dialyzed against deionized water using dialysis tubing (molecular weight cutoff 3.5 kDa) for 48 h to remove the unreacted materials and other impurities and was subsequently freeze-dried to obtain a cotton-like powder.

A Varian Unity 300 MHz spectrometer was used to obtain ^1^H NMR spectra, and D_2_O was used as the solvent. Fourier transform infrared (FTIR) spectral studies were performed using a Nicolet/Nexus 670 FTIR spectrometer between 4000 and 400 cm^−1^ at a resolution of 2 cm^−1^. All powder samples were compressed into KBr pellets in the FTIR measurements. The X-ray diffraction (XRD) pattern was obtained with a D8 Focus (Bruker Corporation, Bremen, Germany) under Cu-Ka radiation (λ = 1.5406 Å) with a scanning speed of 4° min^−1^ and a step of 0.02° (2θ) in the range from 5° to 50°. Thermogravimetric analyzes (TGAs) were performed using TA instrument (SDT Q600, New Castle, PA, USA) with a heating rate of 10 °C·min^−1^. The temperature was increased from room temperature to 400 °C.

### Preparation and characterization of drug-loading system eye drops

The novel ECZ-loading system eye drops (experimental group, E group) were prepared as follows: a solution of ECZ (0.060 g) and malic acid (0.028 g) in acetone was placed in a flask and dried under nitrogen. Then, the conjugated polymer (1.4002 g) and 20 mL of 0.9% of physiological saline were added. The mixture was shocked and equilibrated at room temperature (25 °C) for 48 h. To eliminate the unloaded drugs, the solution was purified by centrifugation at 3000 rpm, and the upper solution was collected. The solutions were lyophilized to obtain a powder form, and the loading content of the solubilized ECZ was determined by HPLC after soaking with methanol.

Next, 0.3% of ECZ suspension eye drops (control group, C group) was prepared by adding 0.0602 g of ECZ to 10 mL of 0.9% of physiological saline and holding it for at least 20 min in an ultrasonic bath. The particle size was determined to be <25 µm by light microscopy in accordance with the Chinese Pharmacopeia. Ten milliliter of 0.2% of sodium hyaluronate (Shandong Freda Biopharm Co., Ltd., China) solution in physiological saline was then mixed with the formulation. Finally, an eye drop suspension with a pH of 6.26 and an osmotic pressure of 254 mOs·mol/kg were acquired.

### Determination of drug-loading content and *in vitro* drug release

The ECZ-loading content was defined as the weight percentage of drug in the NDDS eye drops. To determine the drug-loading content, pre-weighed freeze-dried power was soaked with methanol. The quantitative determination of ECZ content was performed using an HPLC method. The ECZ concentration was determined in all samples using HPLC (Alliance 2695, Waters, Milliford, MA, USA). A reverse-phase column (X Bridge TM, C18, 3.5 μm, 3.0 × 150 mm, Waters) was used for chromatographic separations. The mobile phase consisted of a mixture of phosphate-buffered (5 mmol/L) aqueous solution and methanol at a ratio of 39:61 (the pH was adjusted to 3.5 using phosphoric acid). The flow rate was 0.6 mL/min, and the column oven was heated to 40 °C. The determination was performed at 225 nm with a sample injection volume of 20 μL. The ECZ amount was calculated based on a calibration curve set up for ECZ in the same mobile phase. The drug-loading content (DLC, wt %) was calculated according to the following formula: DLC = (amount of loaded drug/amount of drug-loaded complexation) × 100%.

The *in vitro* drug-release behavior of the drug-loading system was investigated using a previously published bulk equilibrium reverse dialysis bag technique. Briefly, a pre-prepared ECZ-loading system eye drop was directly placed into a glass apparatus containing 400 mL of a simulated tear fluid solution as the release medium, in which numerous dialysis sacs (MW cutoff: 8000) containing 1 mL of the same simulated tear fluid were previously immersed. These sacs were equilibrated with the sink solution for approximately 30 min prior to experimentation. The release study was performed at 35 °C in an incubator shaker (Kuangbei Industry (Kuangbei Industry Co., Ltd, Shanghai, China). At predetermined time intervals, the dialysis bags were withdrawn and replaced with fresh release medium (1 mL). The contents of free drug were assayed using HPLC analysis. The cumulative amount of released drug was calculated based on a calibration curve of ECZ, and the percentage of drug release from the drug-loading system was plotted against time. The release experiments were conducted in triplicate.

### Animals

Male Japanese white rabbits weighing 1.65–2.45 kg were purchased from Henan Kangda Laboratory Animals Co., Ltd. (China) and were 3 months of age. C57BL/6 mice were purchased from Beijing Vital River Laboratory Animal Technology Co., Ltd. (China) and were 4 months of age. All animals were free of clinically observable ocular surface disease and were of a specific pathogen-free grade. All animal care and experimental protocols were approved by the Ethical Committee of Experimental Animal Care of Henan Eye Institute and complied with National Institutes of Health guidelines. All procedures in the study conformed to the Association for Research in Vision and Ophthalmology Statement for the Use of Animals in Ophthalmic and Vision Research.

### Ocular irritation studies

The ocular irritation effects of ECZ-loading eye drops were evaluated according to the Draize eye test with slight modifications (Wilhelmus, 2001). Test substances (experience group for ECZ-loading eye drops, negative control for saline solution, and positive control for ECZ·HNO_3_ suspension) were administered to the cornea in the left eye five times with a dose of 50 μL each time for 20 min (5-min intervals). The right eye served as a control and was treated with distilled water. At the end of the treatment, six observations were performed to evaluate the ocular tissues at time points of 0.5, 1, 2, 3, 4, and 6 h. The cornea, conjunctiva, and iris were graded using scoring criteria.

### *In vivo* pharmacokinetic studies in rabbits

Seventy-nine healthy Japanese white male rabbits, weighing approximately 1.65–2.45 kg, were divided into four groups at random in a predetermined design; each group was used in accordance with the guidelines of the Chinese Animal Administration and the guidelines of the Association for Research in Vision and Ophthalmology Statement for the Use of Animals in Ophthalmic and Vision Research. According to the predetermined design, the rabbits’ central corneal epithelium was debrided using a trephine with a diameter of 13 mm. Two of the four groups were used as E group, and the other two groups as C group. A dose of 50 µL was instilled into the lower cul-de-sac of each eye of every rabbit, respectively. Aqueous humor was withdrawn with a 26-G needle attached to a disposable syringe at a predetermined time point after a single instillation of the drug formulation. Corneal samples were harvested with surgical scissors and knives. All corneas were rinsed with saline, gently wiped, weighed, and stored in preweighed glass tubes. All samples were stored at –60 °C until used for extraction.

### Tissue extraction

*Aqueous humor*: first, 100 µL of aqueous humor was transferred into a glass test tube, and 100 µL of phosphate buffered saline (PBS) was added. The mixture was vortexed, and 2 mL of ethyl acetate was added. The mixture was vortexed for 2 min and then centrifuged at 3000 rpm for 5 min. The upper organic layer was transferred to another cone glass test tube and evaporated to dryness under nitrogen gas. The residue was reconstituted in 100 µL of methanol, and a 20-µL aliquot of each supernatant was directly injected into the HPLC for analysis.

*Corneas*: the cornea was thawed at room temperature and cut into small pieces with scissors. Then, 100 μL of methanol was added, and the corneal pieces were soaked for 24 h and centrifuged for 10 min at 3000 rpm. The supernatant was transferred to a sample vial for HPLC analysis.

### Assay of ECZ in the cornea and aqueous humor

Quantitative determination of ECZ was performed using an HPLC method. The mobile phase consisted of a mixture of phosphate-buffered (5 mmol/L) aqueous solution and methanol at a ratio of 43:57 for cornea detection and 41:59 for aqueous humor (the pH was adjusted to 3.5 using phosphoric acid). Both flow rates were 0.7 mL/min, and the column oven was heated to 50 °C. The determination was performed at 225 nm. The retention time of ECZ was approximately 7.7 min for corneas and 6.5 min for aqueous humor.

The amount of ECZ in the samples was determined by comparison with appropriate standard curves. The validated calibration curve range for aqueous humor was 0.05–2.0 µg/mL with a correlation coefficient (*r*) of 0.9996; for soaked corneal samples, the range was 0.1–5.0 µg/mL with a correlation coefficient (*r*) of 0.9949. The minimal quantitatively detected concentration was 0.05 µg/mL for aqueous humor and 0.1 µg/mL for corneal soaks. To determine the recovery and intra- and inter-day reproducibility, 0.1, 0.5, and 2.0 µg/mL ECZ in aqueous humor and corneal soaks were measured by assaying five replicates at each concentration level (three levels in the low, intermediate, and high concentration ranges) in three separate analytical runs. Quality control (QC) solutions were prepared with blank aqueous humor or soaked corneal samples as described above.

### Pharmacokinetic and statistical analyses

The ECZ concentrations in the aqueous humor and cornea were plotted vs. time after instillation. The *C*_max_ (peak concentration) and *T*_max_ (peak time) were obtained. AUC_0–_*_t_* [area under the concentration-time curve between 0 and 360 min (ICE) or 240 min (debrided corneal epithelium, DCE)] and *t*_1/2_ (elimination half-life) were calculated using DAS 2.1.1 software.

An unpaired *t*-test (two-tailed) was used to determine the level of significance between groups. Differences were considered statistically significant at *p* < .05.

### *In vivo* 3D imaging

Eye drops with dual fluorescent labeling with Cy5 and C6 vector system were prepared without varying the preparation conditions or procedure. To eliminate unlinked and unloaded fluorescent molecules, the solution was purified by successive rounds of dialysis and centrifugation. The mice were anesthetized via an intraperitoneal injection of pentobarbital sodium (85 mg/kg body weight) (Sigma-Aldrich). A homemade plastic eye cup was mounted on the upward-facing eye and was sealed with Vaseline (Zhengzhou Paini Chemical Reagent Factory, China). Next, 1 mg/mL of dual fluorescently labeled vector system eye drops was instilled into the eye cup to allow for the clear imaging of the corneal layers. The dye solution was left for half an hour before being removed. The cornea was then rinsed three times with 0.9% of saline water, and *in vivo* 3D imaging was immediately performed under a two-photon laser scanning microscope. Two-photon imaging was performed using a Zeiss LSM 780 NLO fluorescence microscope system (Carl Zeiss MicroImaging GmbH, Jena, Germany). The assay was performed as previously reported (Li et al., 2015). An excitation wavelength of 700 nm was used. The emission wavelengths of C6 and Cy5 are 500–550 and 640–710 nm, respectively.

### *In vitro* antifungal activity

*Test isolates*: one clinical strain of *Fusarium solani* that was isolated from FK patients was tested. Isolates were passaged twice at an interval of 5–7 days at 28–30 °C. The ECZ strain was tested three times on the same day. Conidia of the isolates were obtained from fresh cultures. QC isolates: ATCC 6258 (*Candida krusei*) was used as a QC isolate in every batch tested.

*Disc diffusion method***:** the DD method is an additional recognized technique to determine the *in vitro* susceptibility of filamentous fungi to various drugs according to CLSI M51-A guidelines. The disc concentration (10 µg/disc) was based on preliminary experiments in which blank discs were impregnated with 10 µL of suspension containing the drug at 1 µg/mL. Specifically, RPMI-1640 agar plates were inoculated with a suspension of 10^6^ spores by swabbing the agar surface. After the plates were allowed to dry, sterile paper discs containing the corresponding drug (ECZ or natamycin) were placed on the agar surface. The diameters (in mm) of the zone of complete inhibition were determined after 48 h of incubation at 28 °C.

*Microdilution methods*: A broth microdilution method was used according to CLSI guidelines (M38-A). The drugs (ECZ and voriconazole) that were used were prepared as outlined in CLSI document M38-A. The endpoints were determined by a colorimetric method using the TransDetectTM Cell Counting Kit (Sigma-Aldrich, St Louis, MO, USA). The rate of inhibition was calculated by comparing the OD at 550 nm (OD550) of the wells with that of the drug-free control based on the following equation: OD550 of the drug-free well-OD550 of wells that contained the drug/OD550 of the drug-free well ×100%. The minimal inhibitory concentration (MIC) was considered the lowest concentration of drug showing at least a 50% of reduction in OD compared with the growth control well.

## Results and discussion

### Conjugating polymers with precisely tuned degrees of conjugation

The synthetic route for conjugated polymers is shown in Figure S1. CD first underwent carboxymethyl functionalization, followed by attachment to the CS chain through an amide reaction between the carboxyl groups of CDs and amino groups of CSs. To confirm the successful synthesis of the chitosan-conjugated-cyclodextrin polymer (CS-c-CD), nuclear magnetic resonance (NMR) spectroscopy, FTIR spectroscopy, XRD, and thermogravimetric analyses (TGAs) were performed. Figure S2 shows the ^1^H NMR spectra of CS-c-CD. The CS-c-CD spectra showed characteristic peaks of 3.3–4.0 ppm with integrated CS at 3.6–4.0 ppm and CD at 3.3–3.8 ppm. In the spectra, the anomeric protons of the α-D-glucopyranosyl residues in the CD moiety at 4.9–5.0 ppm and residues in the CS skeleton at 2.7–2.9 ppm were observed. In addition, the resonance peaks at 1.8–2.0 ppm were attributed to the methyl groups of CS and methyl group methylene CD-COOH. These results confirmed that CD was conjugated onto the CS backbone, in agreement with previous reports (Prabaharan & Jayakumar, 2009). From the area ratio of the signals for these anomeric protons (the CD moiety at 4.9–5 ppm *vs* the CS residues at 2.7–2.9 ppm), the degree of substitution (DS) of CDs onto CSs was estimated to be approximately 27–79% (Figure S2). The conjugation of CD onto CS was also confirmed by FTIR spectroscopy (Figure S3). In the FTIR spectra, after the carboxyl functionalization reaction of CD, a new peak for CD-COOH at approximately 1704 cm^−1^ was observed, which was assigned to the stretching vibration of the carbonyl group (Li et al., 2014a). The CS-c-CD spectrum exhibited increased intensity at 3500 cm^−1^ compared to CS, which was attributed to the higher number of –OH groups in the conjugating polymer. Furthermore, the characteristic peak of the β-pyranyl vibration of CS at 898 cm^−1^, the characteristic peak of the α-pyranyl vibration at 951 cm^−1^ and the aliphatic C-H stretch of CD at 2930 cm^−1^ were observed in the CS-c-CD IR spectra. However, the stretching vibration of the carbonyl group shifted to 1740 from 1704 cm^−1^. The peak intensity at 1520 cm^−1^, which presented –NH_2_ deformation, was dramatically weakened compared to that in the CS spectrum, particularly at high DS (such as 79%). These results indicate that the amine groups of CS were partly conjugated by CD (Li et al., 2014b; Huang et al., 2016). The results of XRD and TGA also confirmed the successful conjugation of CD onto CS (Figure S4). All the relevant diffraction peaks disappeared in the conjugated polymer, which existed in CS, and only an amorphous halo was found (Figure S4(A)). The lack of crystallinity in the conjugated polymer may be due to the loss of regularity throughout the CS’s backbone after the conjugating of CD. The TGA of CS-c-CD showed a single decomposition peak at a temperature similar to that of CS, indicating that the degradation was mainly controlled by this polymer (Alamdarnejad et al., 2013).

### Formulations for drug solubilization and increasing viscosity

ECZ is a potent, topically applied broad-spectrum antifungal agent; however, its very low water solubility limits its therapeutic applications. The water solubility and antifungal activity of ECZ can be improved by complex formation with α- and β-CD. Optimal drug solubilization has also been obtained in ternary systems of ECZ/α-CD/malic acid (Faucci et al., 2000). In the present system, drug-loading capacity was significantly improved by utilizing the ternary complex strategy; as a result, higher DLC (0.3%), more than 600-fold greater (3 vs. 5 µg/mL), was achieved using the NDDS. The DLS was enhanced by increasing the polymer concentration and DS of CD onto CS or by adjusting the molar ratio of the three components. A higher DS and polymer concentration in the NDDS increased drug loading by permitting the inclusion of more drug molecules in the large number of α-CD hydrophobic cores of the polymer via hydrophobic-hydrophobic interactions. Moreover, the simultaneous presence of the third component of hydroxyl acid and α-CD had an additive effect, which increased the aqueous solubility of ECZ more effectively than binary complexation (Faucci et al., 2000). The optimal drug solubilization was obtained at an approximately 1:1:1 molar ratio of ECZ:α-CD:malic acid (Mura et al., 2001). The NDDS formulations had a pH of 5.40 ± 0.06 and isotonic value of 250 ± 5 mOs·mol/kg; these values are within the acceptable normal ranges and are unlikely to cause irritation after application. Mucoadhesive properties are a key factor for ocular topical formulations to prolong the residence time of drugs or pharmaceuticals at specific delivery sites. CS is a widely known mucoadhesive polymer due to its strong interaction with the mucin layer by numerous inter-molecular hydrogen bonds and electrostatic interactions with the negatively charged residues in mucin (Sosnik et al., 2014). The present formulations had a viscosity of 34.5 cP, far higher than the viscosity of water (approximately 1 cP), and can therefore prolong the residence time of drugs on the ocular surface and improve drug bioavailability.

Figure S5 demonstrates the cumulative release profiles for the ECZ-loaded delivery system as a function of time. The release profiles showed a biphasic release mode: an initial rapid release in the first 12 h followed by a slow and steady release over a prolonged time period of up to several hours. The drug release from the system reached 48.8% in 12 h. Approximately 30% of the drug was released in approximately 1 h. The abrupt release in the beginning was mainly due to the drug remaining at the surface without being efficiently entrapped within the polymeric vectors. After the initial period, the drug was released in a slow, sustained manner, which probably occurred via a diffusion-controlled mechanism (Prabaharan & Jayakumar, 2009). The *in vitro* release results showed that the NDDS might be suitable for ECZ corneal delivery with the rapid attainment of the effective drug concentration and sustained release allowing the dosage frequency to be decreased.

### Reducing ocular irritation

**Figure 2.**
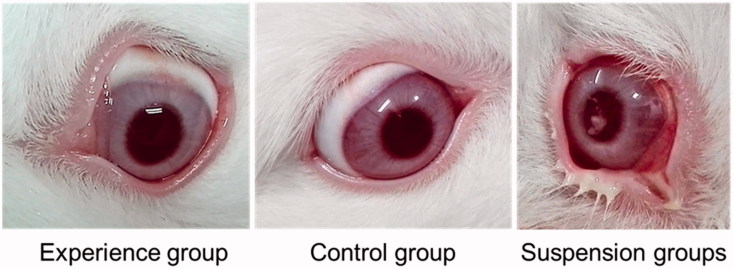
Representative pictures of the *in vivo* rabbit ocular irritation test; the experience group received econazole (ECZ)-loaded NDDS eye drops, the control group received saline (control group), and the suspension group received ECZ nitrate suspension.

Eye drops are used directly on the corneal surface; consequently, ophthalmic irritation is a key factor affecting ophthalmic drug development and clinical use. CDs have been applied to decrease drug irritation via the formation of inclusion complexes (Loftssona & Järvinen, 1999). To reduce irritation and improve solubility, we designed and fabricated a conjugated polymer as a drug delivery vector. Evaluation of irritation caused by the NDDS formulation according to the Draize test in rabbits *in vivo* revealed no macroscopic signs of irritation in the cornea, conjunctiva or iris. Additionally, the scores for corneal opacity, iris hyperemia, conjunctiva swelling, and discharge were always grade zero in all observations, whereas the suspension group exhibited intense irritation (Figure 2). Hence, the NDDS based on the CS-c-CD polymer effectively decreases irritation from the antifungal drug ECZ, enabling potential clinical applications.

### Pharmacokinetics of ECZ in rabbit eyes

To treat FK, a high concentration of the antimycotic drug in the cornea and aqueous humor is desired. However, ECZ is poorly soluble in water, and a vehicle is necessary to increase the solubility of ECZ for administration in an aqueous eye drop solution. An HPLC method for analyzing ECZ in samples of both aqueous humor and cornea was successfully established, with clear separation of ECZ from other substances. There was a linear relationship between the ECZ concentration and integrated spectrum peak area. The linear regression equations were fitted over the entire calibration range of 0.05–2.0 µg/mL for aqueous humor and 0.1–5.0 µg/mL for corneal soaks with good linearity and correlation coefficients *r* > 0.99. The recovery and intra- and inter-day reproducibility results are shown in Table S1. The mean recoveries from the aqueous humor and cornea were >91% for each group of samples, thus validating the calibration curves. There were no significant differences in ionization efficiency between the working solution and tissue extract.

**Figure 3.**
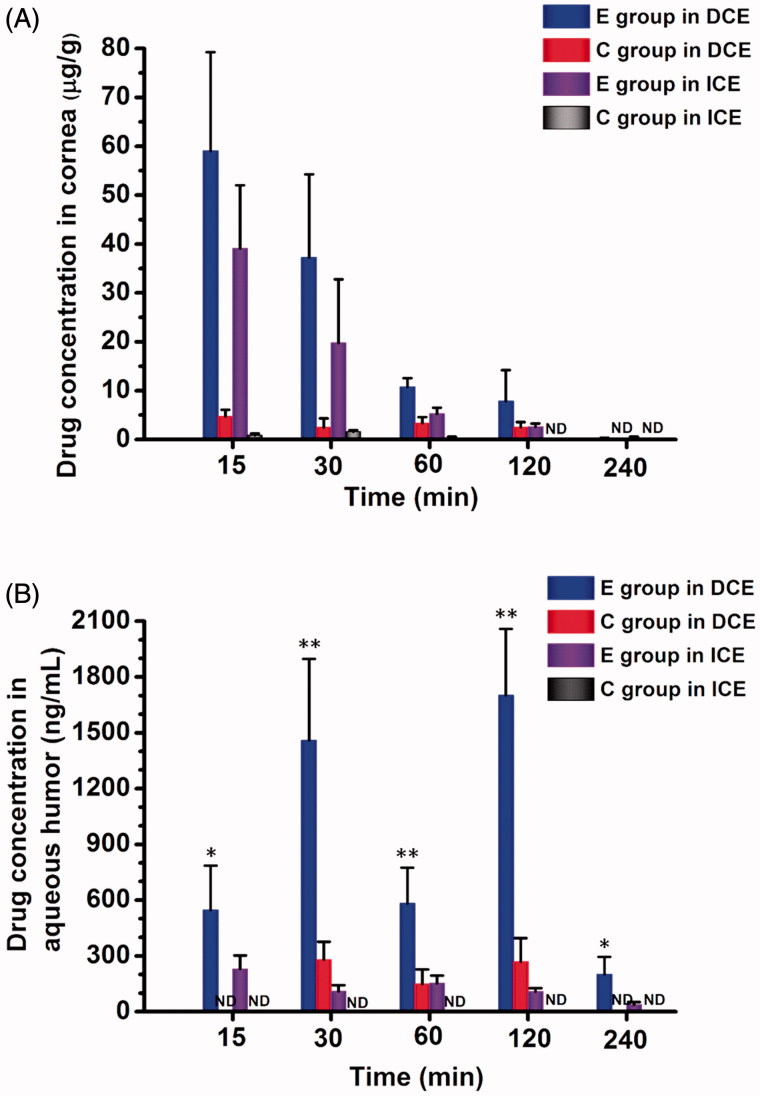
Pharmacokinetics studies in rabbit eyes after a single instillation of either formulation. Econazole (ECZ) concentration time profiles in (A) cornea and (B) aqueous humor of rabbits. The drugs included 0.3% of ECZ solution eye drops (E group) and 0.3% of ECZ suspension eye drops (C group) with a single dose of 50 µL. Values are given as the mean ± SD (*n* = 6), ND, not detected; DCE, debrided corneal epithelium; and ICE, intact corneal epithelium. ∗*p* < .05 for E group with DCE versus with ICE; ∗∗*p* < .01 for E group with DCE vs. C group with DCE and E group with ICE (unpaired *t*-test).

The method was then applied to assay changes in ECZ concentrations in the aqueous humor and cornea. The mean ECZ concentrations in the aqueous humor and corneas at different time intervals after administration are shown in Table S2 (ICE) and Table S3 (DCE). The time course of ECZ concentrations in the aqueous humor and corneas following the administration of different formulations is displayed in Figure 3.

In ICE rabbits, after a single dose of topical eye drops of ECZ solution in the NDDS (E group, 0.3%), ECZ levels in the cornea were significantly increased at 5–360 min compared with those after the administration of ECZ suspension eye drops (C group, 0.3%) (*p* < 0.01). In contrast, the ECZ levels in the cornea were undetectable at 90 min after topical application in C group (Figure 3 and Table S2). The highest ECZ levels in the cornea (62.31 ± 14.05 μg/g) were achieved at 5 min after administration in E group and were 59 times greater than that in C group. The ECZ levels in the aqueous humor were measurable for E group, and the peak concentration, 229.85 ± 72.25 ng/mL, was observed at 15 min, whereas ECZ was not detectable at any time points in C group. These results indicate that the NDDS can elevate drug concentrations in the aqueous humor and cornea, thus enhancing ECZ penetration across the cornea.

In DCE rabbits, after a single administration to E group, the maximum ECZ concentrations in the cornea and aqueous humors were 59.04 ± 20.21 μg/g at 15 min and 1701.85 ± 356.47 ng/mL at 120 min, 12.4 and 6.3 times greater than in C group, respectively (Figure 3 and Table S3). The ECZ levels in the cornea and aqueous humors were measurable at all time points in E group, but were undetectable in the cornea at 240 min and aqueous humors at 5 min and 240 min in C group. These results indicate that the NDDS can enhance the corneal penetration of ECZ.

Interestingly, two ECZ concentration peaks >1495 ng/mL were observed in the aqueous humor at 30 and 120 min. These values may be attributed to differences in the diffusion of the small-molecule drug and the macromolecular vector. The peak at 30 min is due to rapid transmission across the cornea of ECZ released from the system, whereas the peak at 120 min is due to slow passage of the drug-loaded system comprising drug and the large molecular conjugated polymer. These two peaks at 30 min and 120 min were also observed in C group at lower concentrations and they did not differ significantly from one another. These peaks were not observed in ICE rabbits because the tight junctions in the corneal epithelium prevent the entry of macromolecules, including CD (Loftssona & Järvinen, 1999).

Furthermore, after single-dose topical administration, the ECZ concentration was higher in DCE rabbits than in ICE rabbits at each time point (Figure 3 and Tables S2 and S3), indicating that corneal permeability increases in the absence of the corneal epithelium. The ECZ concentration in the aqueous humor was also significantly increased at each time point in DCE group compared with that in ICE group (*p* < 0.05), with increases of approximately 13.7- and 15.7-fold higher at 30 and 120 min, respectively (Tables S2 and S3). This increase is likely attributable to passage of the water-soluble drug-loading system through the hydrophilic stroma when the corneal epithelium was debrided (Li et al., 2015). These results revealed that the NDDS enhances corneal penetration and the maintenance of long-term, higher drug concentrations in the aqueous humor, thus permitting drug delivery to the ocular posterior segment.

The key pharmacokinetic parameters of ECZ in the aqueous humor and cornea, such as the area under concentration-time curves (AUC_0–_*_t_*), peak concentration (*C*_max_), peak time (*T*_max_), and elimination half-life (*t*_1/2_), were calculated based on the administered concentration and are shown in Table S4. As indicated in Table S4, the estimated ocular relative bioavailability (area under the concentration-time curve between 0 and *t*_min_, AUC_0–_*_t_*) in the cornea was 29-fold (ICE, *t* = 360 min) and 5.7-fold (DCE, *t* = 240 min) greater in E group than in C group. Taken together, these results indicate that the NDDS enhances the drug’s corneal penetration in the presence of both ICE and DCE.

### *In vivo* evaluation of corneal penetration of NDDS

The vector in our NDDS is a hydrophilic and mucoadhesive macromolecule that should be easily retained on the ocular surface and should enhance the corneal penetration of drugs. To verify this potential, *in vivo* fluorescence imaging with dual fluorescent labeling was performed to monitor corneal penetration in mice. A hydrophobic dye, coumarin-6 (C6), was used as a model drug. The visible green spots of C6 fluorescence correspond to released C6, mimicking the release behavior of ECZ. The polymer was labeled with cyanine5-NHS (Cy5) to monitor the transmission behavior of the polymeric vector in the cornea.

Two-photon laser scanning microscopy is a new, powerful technology for studying live cells and tissues as well as for in-depth imaging of thick biological samples and long-term observations. In the present study, two-photon laser scanning microscopy was employed to monitor the *in vivo* penetration behavior of a fluorescent molecule in the murine cornea. Although numerous reports have discussed the ocular penetration of drugs, the present study is the first to use dual fluorescence labeling to clearly visualize the penetration behaviors of drugs into the mouse cornea *in vivo* in real time.

**Figure 4.**
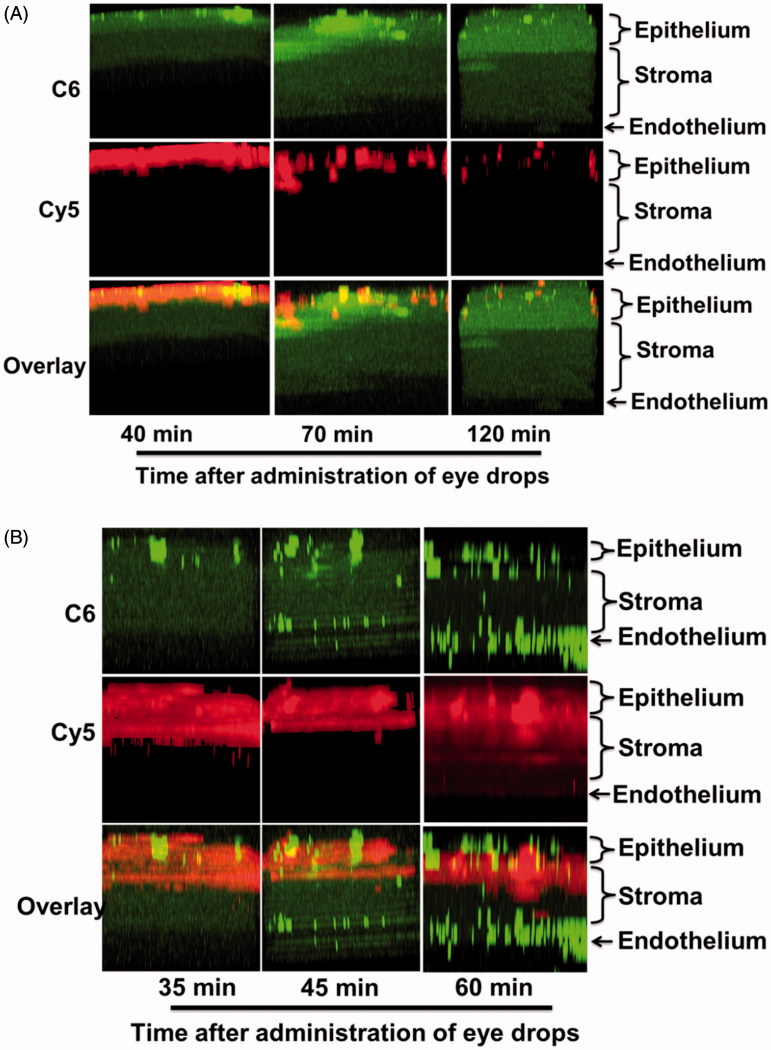
Representative *in vivo* two-photon microscopy corneal cross-section images at different times after topical administration of the dual fluorescently labeled formulation of Cy5 and C6 in c57Bl/6 mice. The tests were performed under (A) ICE and (B) DCE (magnification, 100×).

**Figure 5.**
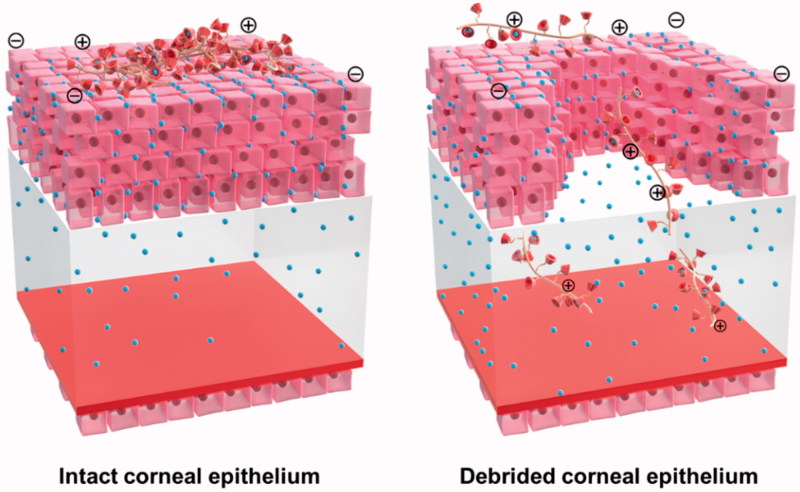
Schematic illustration of the corneal penetration behavior of NDDSs after topical administration under ICE and DCE *in vivo*.

The time-dependent corneal penetration behaviors were estimated after topically administering the NDDS, and the permeation mechanisms are discussed. In ICE mice, the polymeric vector was localized only on the epithelium surface, but the C6 model drug was gradually released from the system and diffused to the stroma and endothelium over an extended time of 120 min (Figures 4(A) and S6(A)). The results are in agreement with previous reports that macromolecules (including CD) do not penetrate biological membranes (such as the epithelium layer of the corneal barrier) (Loftssona & Järvinen, 1999). In the present NDDS, enhanced corneal penetration of the drugs is achieved by prolonging the retention time on the ocular surface and sustained release to ensure a high concentration of the dissolved drug on the precorneal area (Figure 5).

For DCE, the permeation behavior was slightly different. In addition to the diffusion of free drug from the surface layer of the cornea to the deep layer (green dispersion spots), the loaded drug (green highlighted spots), together with the polymeric vector (red spots), reached the stroma and endothelial surface, the deepest part of the cornea in contact with the anterior chamber (Figures 4(B) and S6(B)). In this case, the corneal epithelium was incomplete, and these tight junctions were destroyed. Consequently, the hydrophilic drug-loaded polymer carrier crossed the hydrophilic stroma, a major barrier to hydrophobic molecules. This study clearly demonstrates that the drug-loaded NDDS can accumulate in all corneal layers to release ECZ and permit drug action (Figure 5). These observations may also explain the double-peak phenomenon in the pharmacokinetics test in DCE group due to the discrepant diffusion of the small-molecule drug and macromolecular vector. In brief, these *in vivo* studies indicated that the NDDS can enhance corneal penetration of both ICE and DCE, in agreement with the results of the pharmacokinetic studies.

### *In vitro* evaluation of the antifungal activity of NDDS

**Figure 6.**
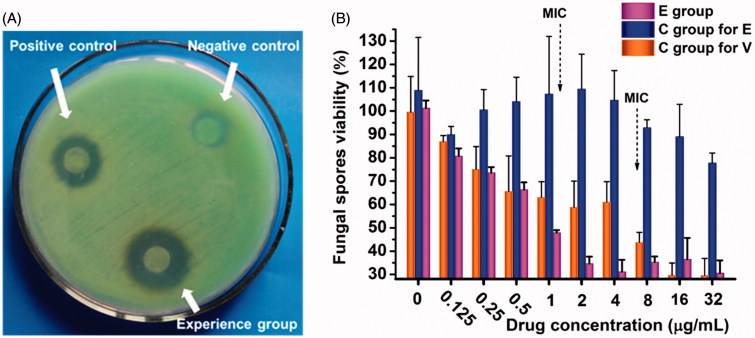
*In vitro* drug sensitivity of fungi using different methods. (A) Disc diffusion method. Experience group, econazole (ECZ) loading in NDDS; negative control, ECZ suspension; positive control, natamycin eye drops. (B) Microdilution method. E group, ECZ solution eye drops; C group for E, ECZ suspension eye drops; and C group for V, voriconazole solution eye drops. The curves represent the inhibition of fungal spores by ECZ and voriconazole.

After acquiring a drug delivery platform with optimal corneal penetration properties, *in vitro* antifungal activity was evaluated. We performed antifungal assays using the disc diffusion and microdilution methods to determine whether the NDDS formulation influences the antifungal activity of ECZ. The results are presented in Figure 6. Compared to the controls, a larger inhibition zone diameter against the *Fusarium* genus was observed for the NDDS, indicating that the NDDS has good antifungal activity (Figure 6(A)). Additionally, the MIC values of ECZ and voriconazole were measured using serial microdilution methods, and different ECZ formulations were compared. The MIC of ECZ in the NDDS against *Fusarium solani* was 1.0 μg/mL, in agreement with a previous report, whereas the MIC of voriconazole was 8.0 μg/mL (Figure 6(B)) (Oji & Clayton, 1982). The antifungal abilities of ECZ in the NDDS were significantly enhanced compared with those of the suspension. The improved antimycotic activity in the NDDS might be due to an increase in affinity between the NDDS and the fungi, and the superior ability of the inclusion complex to cause ECZ supersaturation is in agreement with previous reports (Pedersen et al., 1998). These results suggest that the NDDS enables the high level of antifungal activity of ECZ.

As a mucoadhesive polymer, CS has good potential for ocular drug delivery by increasing the viscosity of eye drop solutions and prolonging the retention time of the drug on the ocular surface after topical administration (Ali et al., 2016). These effects of CS are due to the strong interaction with the mucin layer by inter-molecular hydrogen bonds and electrostatic interactions with the negatively charged sialic acid and sulfate residues in mucin. CDs are cyclic oligosaccharides with a hydrophilic exterior surface and a hydrophobic interior cavity (Crini, 2014). Due to their low toxicity and low immunogenicity, CD is used in pharmaceutical formulations to increase drug solubility and stability, control drug release, alleviate local, and systemic toxicities and improve drug permeability across biological barriers. In ocular drug delivery, CD can increase the water solubility of a drug and reduce local irritation (Zhang & Ma, 2013). In general, CD molecules do not penetrate biological membranes but enhance drug corneal penetration and bioavailability by ensuring a high concentration of the dissolved drug in the precorneal region (Sigurdsson et al., 2005). As a proof-of-concept demonstration, we designed and synthesized a conjugated polymer based on CS and CD as a novel ocular drug delivery carrier. This vector combines the favorable qualities of the two building units, resulting in a synergistic effect. The advantages of this vector include the following: (1) increased apparent drug solubility in eye drop solutions; (2) reduced local irritation after topical administration; (3) prolonged drug residence time at precorneal and corneal sites; (4) improved drug-release properties due to controlled-release behavior; and (5) decreased dosage frequency, thus enabling good patient compliance and enhanced corneal penetration after topical administration (Figure 1(B)). Utilizing this polymeric vector, a new formulation of ECZ for topical ocular application was developed.

ECZ is an antifungal drug with excellent antifungal activity; however, there are no appropriate ocular pharmaceutical formulations of ECZ due to its severe irritant properties and low water solubility. Consequently, novel topical ocular drug delivery systems are needed for the treatment of FK using ECZ. In the present work, a new ECZ formulation was developed. The NDDS increased ECZ solubility and reduced irritation via complexation with CD. Notably, the NDDS will have therapeutic effects in all FK processes due to their superior corneal penetration in both ICE and DCE. Other antifungal drugs may work only in the early stage, when the corneal epithelium is debrided, and not in the later stage, due to poor penetration following epithelium repair, leading to treatment failure.

## Conclusion

A new class of ocular drug delivery systems has been developed. This new platform is capable of significantly improving drug solubility and reducing ocular irritation. Pharmacokinetic studies revealed high ECZ concentrations in the cornea and aqueous humor under both ICE and DCE, indicating that the NDDS enhances corneal penetration and improves drug bioavailability. A corneal penetration study performed with dual fluorescence labeling clearly demonstrated the penetration capacity of the NDDS in accordance with the pharmacokinetics results. These findings are important both for understanding the penetration behavior in the cornea after topical administration and guiding improvements in new drug delivery strategies for ocular use. This platform technology is a promising candidate for FK therapy that can also be applied with various hydrophobic drugs to overcome the barriers to the treatment of many other ocular diseases.

## Supplementary Material

IDRD_Li_et_al_Supplemental_Content.pdf
