# Supplementary material for: Fabrication of a drug delivery system that enhances antifungal drug corneal penetration
Source: Drug Deliv. 2018 Apr 16;25(1):938–49. doi: 10.1080/10717544.2018.1461278 (PMC6058611; doi:10.1080/10717544.2018.1461278)
Supplement: IDRD_Li_et_al_Supplemental_Content.pdf [file IDRD_A_1461278_SM0578.pdf]

Supporting Information

Fabrication of a drug delivery system that enhances antifungal drug corneal penetration

Jingguo Li┼*, Zhanrong Li┼, Zhen Liang, Lei Han, Huayang Feng, Siyu He, Junjie Zhang*

Henan Key Laboratory of Ophthalmology and Visual Science, Henan Eye Hospital, People's Hospital of Zhengzhou University, Zhengzhou, P. R. China

Corresponding authors

*J.G.L. Fax: +86-371-65952907; e-mail: lijingguo@zzu.edu.cn.

*J.J.Z. Fax: +86-371-65952907; e-mail: zhangjj66@126.com

┼These authors contributed equally to this work.

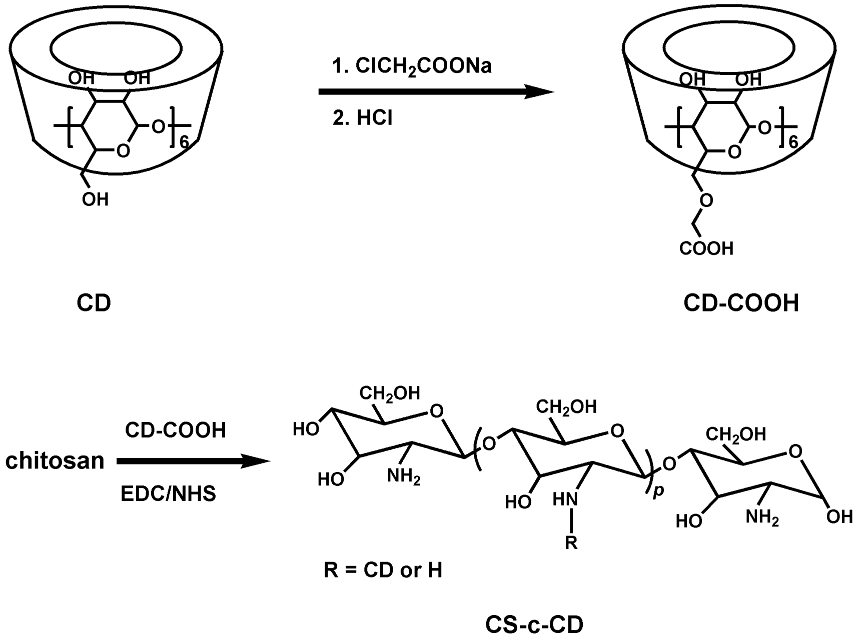

Figure S1. Synthetic route of the conjugated polymer.

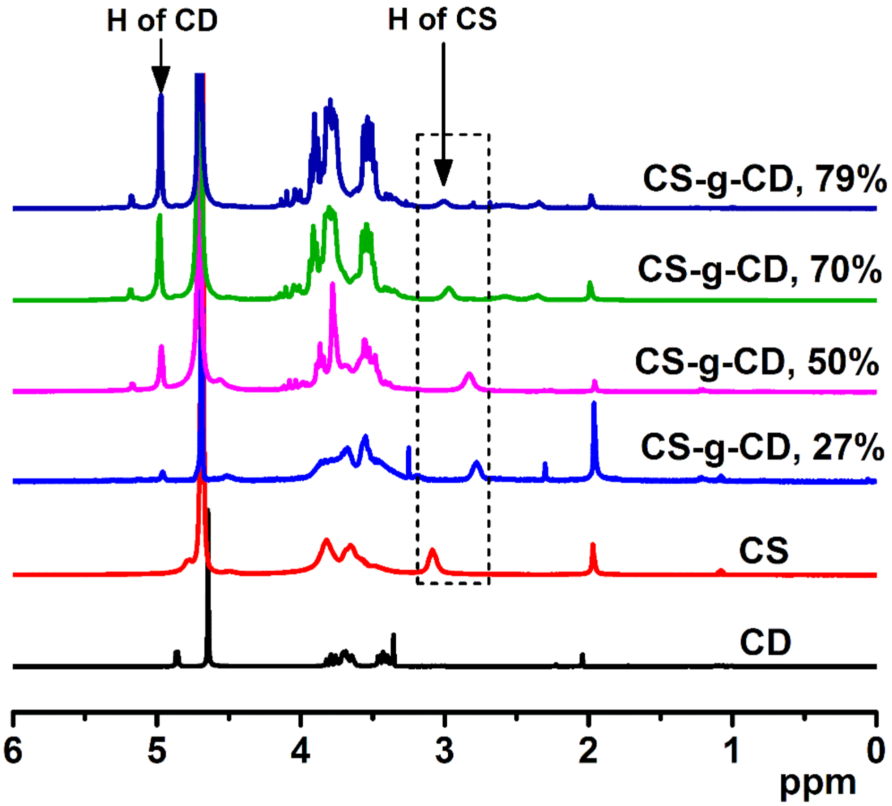

Figure S2. ^{1}H NMR spectroscopy of the conjugated polymers. Spectra in D_2O were recorded on a Varian Unity 300 MHz spectrometer at 25 °C.

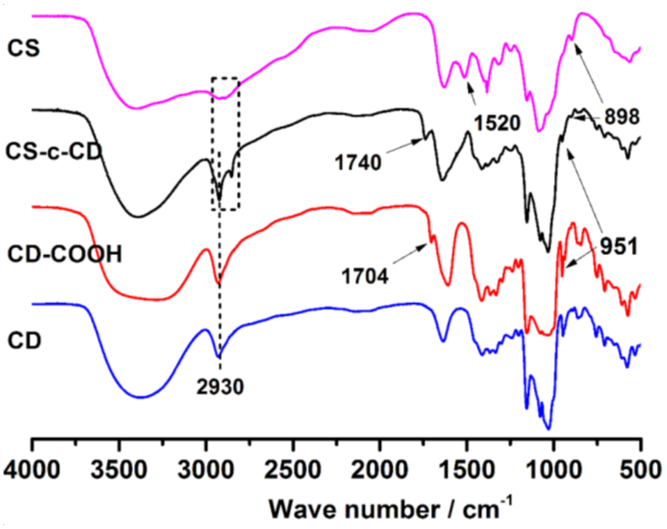

Figure S3. FTIR spectra of the conjugated polymer and its building units.

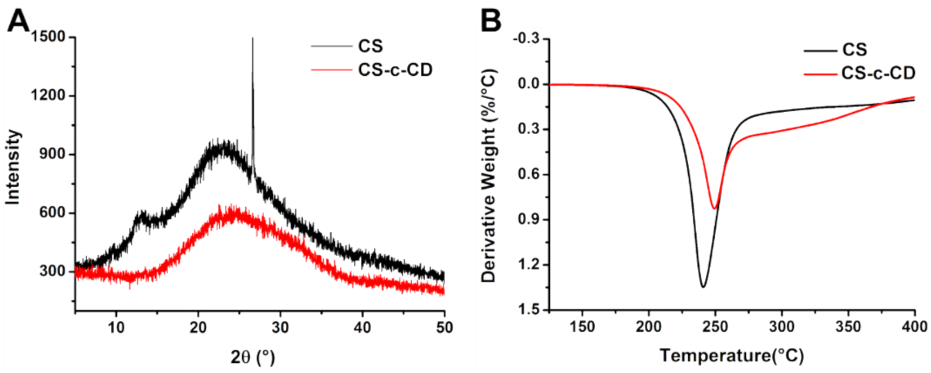

Figure S4. (A) X-ray diffraction patterns of CS and CS-c-CD. (B) Thermogravimetric analyses of CS and CS-c-CD.

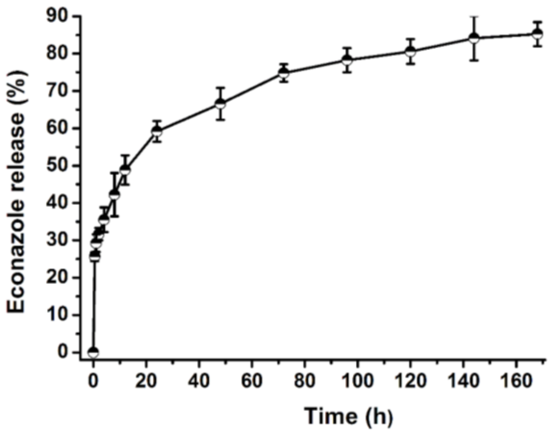

Figure S5. *In vitro* release profile of econazole-loaded system at pH 7.2.

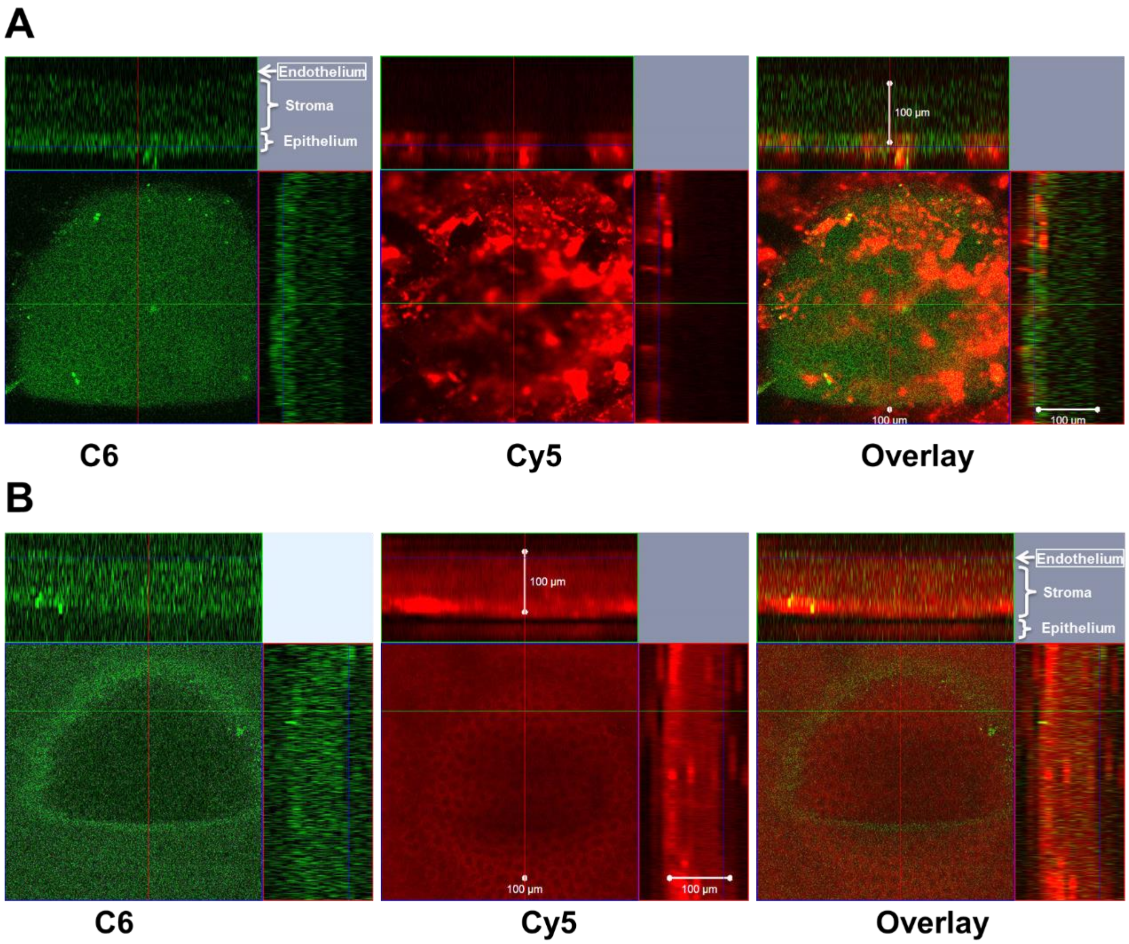

Figure S6. Representative *in vivo* two-photon microscopy images of corneal cross-sections at different times after the topical administration of a dual fluorescently labeled formulation of Cy5 and C6 in C57BL/6mice. Each image shows xy (left lower quadrant), xz (right lower quadrant) and yz (right upper quadrant lower panel) slices through a 3D stack. (A) ICE 90 min after administration; (B) DCE 35 min after administration. According to our previous report,[1] the corneal center thickness in C57BL/6 mice was 116.6 ± 7.6 µm, including 39.9 ± 4.2 µm in the epithelial layer and 76.7 ± 6.0 µm in the stromal and endothelial layers.

Table S1. Results of the recovery and precision of econazole in rabbit cornea and aqueous humor.

tissues	Concentration (μg/mL)	Recovery ($\bar{x}$ ± SD%)[a]	Intraday[c] ($\bar{x}$ ± RSD%)[b]	Interday[d] ($\bar{x}$ ± RSD%)
Aqueous humor	0.1	91.8 ± 2.66	0.092 ± 2.90	0.127 ± 11.02
	0.5	103.5 ± 6.96	0.518 ± 13.44	0.452 ± 3.75
	2.0	108.5 ± 8.30	2.169 ± 3.83	2.06 ± 4.19
Cornea	0.1	103.8 ± 6.63	0.109 ± 6.09	0.104 ± 7.35
	0.5	99.7 ± 7.82	0.499 ± 1.57	0.503 ± 2.19
	2.0	99.3 ± 3.61	1.987 ± 1.81	2.128 ± 2.84

[a] $\bar{x}$: mean values; SD, standard deviation; [b] RSD, relative standard deviation; [c] five replicates at each concentration level; [d] three runs with five replicates at each concentration level over a period of 3 days.

Table S2. Mean concentrations of econazole (ECZ) in the aqueous humor and cornea after a single dose (50 μL) topical administration of 0.3% ECZ solution eye drops (E group) and 0.3% ECZ suspension eye drops (C group) in rabbit eyes with intact corneal epithelium.

Time (min)	Aqueous humor (ng/mL)		Cornea (μg/g)	
	E group	C group	E group	C group
5	159.64 ± 51.55	ND	62.31 ± 14.05**	1.06 ± 0.54
15	229.85 ± 72.25	ND	39.10 ± 12.92**	0.89 ± 0.35
30	109.16 ± 33.38	ND	19.73 ± 12.99**	1.62 ± 0.30
60	152.77 ± 41.61	ND	5.26 ± 1.25**	0.40 ± 0.19
90	85.34 ± 19.97	ND	1.56 ± 0.75	ND
120	108.31 ± 18.19	ND	2.63 ± 0.66	ND
180	63.60 ± 24.63	ND	0.50 ± 0.16	ND
240	37.29 ± 14.49	ND	0.38 ± 0.20	ND
360	51.54 ± 28.03	/	0.11 ± 0.04	/

The values are presented as the means ± SD (n = 6) (SD: standard deviation); concentrations for the E group compared with the C group, ** $p < 0.01$ (unpaired t-test); ND: not detected.

Table S3. Mean concentrations of econazole (ECZ) in the aqueous humor and cornea after a single dose (50 µL) topical administration of 0.3% ECZ solution eye drops (E group) and 0.3% ECZ suspension eye drops (C group) in rabbit eyes with debrided corneal epithelium.

Time (min)	Aqueous humor (ng/mL)		Cornea (µg/g)	
	E group	C group	E group	C group
15	546.80 ± 238.60	ND	59.04 ± 20.21**	4.75 ± 1.36
30	1495.52 ± 438.11**	280.08 ± 96.41	37.24 ± 17.04**	2.51 ± 1.79
60	582.50 ± 191.59**	148.35 ± 79.18	10.78 ± 1.75**	3.36 ± 1.65
120	1701.85 ± 356.47**	269.39 ± 126.8	7.87 ± 6.33*	2.54 ± 1.03
240	200.71 ± 94.91	ND	0.23 ± 0.09	ND

The values are presented as the means ± SD (n = 6) (SD: standard deviation); concentrations for the E group compared with the C group, ∗ $p < 0.05$; ∗∗ $p < 0.01$ (unpaired t-test); ND, not detected.

Table S4. Main pharmacokinetic (PK) parameters of econazole (ECZ) after a single dose 50 µL topical instillation of either eye drops into rabbit eyes with intact corneal epithelium (t = 360 min) or with debrided corneal epithelium (t = 240 min). E group, 0.3% ECZ solution eye drops; C group, 0.3% ECZ suspension eye drops. Each value represents the mean ± SD (n = 6) (SD: standard deviation.

PK parameters			AUC_{0-t} (min·µg/mL)	T_{max} (min)	C_{max} [a]	$t_{1/2}$ (min)
Intact corneal epithelium	Aqueous humor	E group	28.83	15	229.85 ± 72.25	78
		C group	/	/	/	/
	Cornea	E group	1797.54	5	62.31 ± 14.05	69.48
		C group	61.95	30	1.62 ± 0.30	/
Debrided corneal epithelium	Aqueous humor	E group	233.27	120	1701.9 ± 356.3	142.2
		C group	/	30	280.08 ± 96.41	/
	Cornea	E group	2929.86	15	59.04 ± 20.21	28.26
		C group	511.8	15	4.75 ± 1.36	39

[a] The units are ng/mL for aqueous humor and µg/g for cornea.

REFERENCES

(1) Zhang, H. M.; Wang, L. Y.; Xie, Y. T.; Liu, S. S.; Deng, X. M.; He, S. Y.; Chen, G. M.; Liu, H.; Yang, B.; Zhang, J. J.; Sun, S. T.; Li, X. H.; Li, Z. J. The Measurement of Corneal Thickness from Center to Limbus in Vivo in C57BL/6 and BALB/c Mice Using Two-photon Imaging. *Exp. Eye Res.* **2013**, *115*, 255-262.
